# Importance of *Dendrobium officinale* in improving the adverse effects of high-fat diet on mice associated with intestinal contents microbiota

**DOI:** 10.3389/fnut.2022.957334

**Published:** 2022-07-28

**Authors:** Xiaoya Li, Na Deng, Tao Zheng, Bo Qiao, Maijiao Peng, Nenqun Xiao, Zhoujin Tan

**Affiliations:** ^1^College of Chinese Medicine, Hunan University of Chinese Medicine, Changsha, China; ^2^College of Pharmacy, Hunan University of Chinese Medicine, Changsha, China

**Keywords:** *Dendrobium officinale*, high-fat diet, intestinal contents microbiota, diversity, dominant bacteria

## Abstract

A growing body of evidence suggests that the disturbance of intestinal microbiota induced by high-fat diet is the main factor causing many diseases. *Dendrobium officinale* (DO), a medicinal and edible homologous Chinese herbal medicine, plays essential role in regulating intestinal microbiota. However, the extent of DO on the intestinal contents microbiota in mice fed with a high-fat diet still remains unclear. Therefore, this study explored the role of intestinal contents microbiota in the regulation of adverse effects caused by high-fat diet by DO from the perspective of intestinal microecology. Twenty-four mice were randomly distributed into the normal saline-treated basal diet (bcn), normal saline-treated high-fat diet (bmn), 2.37 g kg^−1^ days^−1^ DO traditional decoction-treated high-fat diet (bdn) and 1.19 g kg^−1^ days^−1^ lipid-lowering decoction-treated high-fat diet (bjn) groups for 40 days. Subsequently, we assessed the changes in body weight, serum total cholesterol (TC), total triacylglycerol (TG), low density lipoprotein-cholesterol (LDL-C), high density lipoprotein-cholesterol (HDL-C) levels, and the characteristics of intestinal contents microbiota. Results demonstrated that DO exerted the modulating effect on the changes in body weight, TG, TC, LDL-C, and HDL-C levels. Besides, DO decreased the richness and diversity of intestinal contents microbiota, and altered the structure as a whole. Dominant bacteria, *Ruminococcus* and *Oscillospira*, varied significantly and statistically. Moreover, DO influenced the carbohydrate, amino acid, and energy metabolic functions. Furthermore, *Ruminococcus* and *Oscillospira* presented varying degrees of inhibition/promotion of TG, TC, LDL-C, and HDL-C. Consequently, we hypothesized that *Ruminococcus* and *Oscillospira*, as dominant bacteria, played key roles in the treatment of diseases associated with a high-fat diet DO.

## Introduction

With the development of the social economy and improvement of living standards, the human diet has undergone significant changes, and the intake of meat and fried foods that are rich in saturated fat is increasing. Studies have proved that long-term high-fat diets led to a series of chronic metabolic diseases such as obesity, hyperlipidemia, and diabetes (1). Long-term dyslipidemia can cause excessive lipid deposition on the vascular wall, resulting in atherosclerosis and increasing mortality from cardiovascular and cerebrovascular diseases (2). According to the “Guidelines for the Prevention and Treatment of Dyslipidemia in Chinese Adults (2016 Revision),” the number of dyslipidemia in China has reached 430 million, and the incidence of coronary heart disease is increasing by 30% every 10 years (3). Therefore, prevention and treatment of hyperlipidemia are of great significance for prolonging and improving the quality of life.

As the most important digestive organ of the body, the digestion and absorption of nutrients necessary for human growth mainly occur in the intestine. The intestinal microbiota is a complex ecosystem in which trillions of microorganisms have developed together since the birth of the host, depending on the genome, nutrition, and lifestyle, and participating throughout the body in physiology, biochemistry, pathology, pharmacology, and energy extraction and storage (4). A balanced intestinal microbiota contributes to the healthy growth and development of the host, and disorders of the intestinal microbiota are associated with chronic metabolic disorders in the host (5). A prolonged high-fat diet will continue to alter the structure and function of the intestinal microbiota (6). Animal experiments have confirmed that the abundance of *Enterobacteria* in the intestine of hyperlipidemic rats was strikingly increased, while beneficial microbiota such as *Bifidobacteria, Lactobacillus*, and *Enterococcus* was significantly reduced (7). Obesity in rats was significantly enriched in the proportion of Firmicutes, but poor in the Bacteroidetes and Verrucomicrobia (8). Hence, a rational diet is of great value in maintaining the balance of intestinal microbiota and preventing the occurrence of diseases. However, considering that eating habits are difficult to change among people, it is generally unacceptable for patients to correct bad eating habits with drugs, which involves the risk of drug treatment. Therefore, exploring pharmacology and food-based drugs to improve hyperlipidemia may be a good solution.

*Dendrobium officinale* Kimura et Migo, is the second largest genus of Orchidaceae, which was recorded in the 2015 edition of “Pharmacopeia of the People's Republic of China” (9). Modern pharmacological studies have presented that DO has a variety of intestinal health functions, such as protecting intestinal mucosa (10), regulating intestinal immunity (11), enhancing intestinal motility, promoting defecation (12), and modulating intestinal microbiota (13). Yan et al. have elucidated that polysaccharides from DO regulated intestinal micro-environmental homeostasis by decreasing the ratio of Firmicutes-to-Bacteroidetes of mice on a high fat and sucrose diet and upregulating the production of acetate, propionate, and butyrate (14). Our preliminary study in high-fat diet mice suggested that DO adjusted the diversity of the intestinal mucosal microbiota, promoted the abundance of *Ochrobactrum*, and inhibited the abundance of *Bifdobacterium* and *Ruminococcus* (15). With DO being included in the National Health Commission as a medicinal and edible homologous, it also means that more attention has increasingly been paid to the health value of DO (16). Recent studies have informed that the parenteral pharmacological effects of DO, including lowering blood glucose (17), and regulating blood lipid (18), are also closely related to intestinal pharmacological effects, but the main mechanism of action remains unclear.

**Figure 1:**
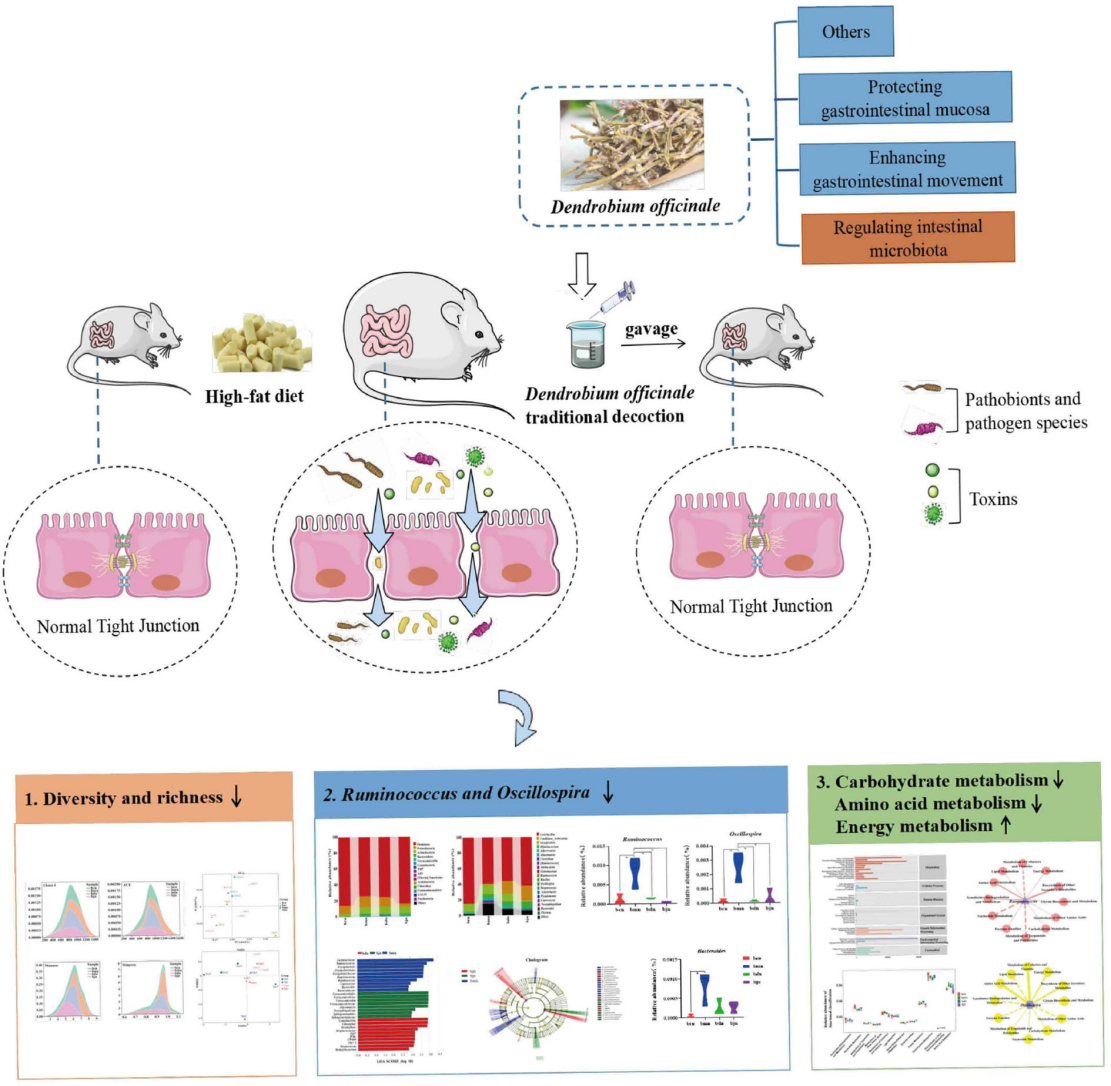
Experimental flow chart.

In this study, we analyzed the effect of DO on the characteristics of intestinal contents microbiota in mice fed with high-fat diet and elucidated the correlation between the dominant bacteria and high-fat diet. Concomitantly, we sought to understand the main mechanism by which DO regulated intestinal contents microbiota in mice fed with high-fat diet. It is expected to provide theoretical support for the health efficacy of DO from the perspective of intestinal microecology and contribute to the experimental basis for the development of DO (Figure 1).

## Materials and methods

### Medicine

Superfine powder of DO was produced from Hunan Longshishan *D. officinale* Base Co., Ltd (Changsha, Hunan, China). The preparation process of ultra-micro powder of DO was as follows: The stems of the DO (Hunan Longshishan *D. officinale* Base Co., Ltd, Changsha, Hunan) were cut off, dried, and crushed with the FW135 Herbal Grinder (Tianjin City Taisite Instrument Co. Ltd., Tianjin) and passed through 100 mesh to obtain the fine powder of DO. Subsequently, 200 g of the fine powder of DO was taken and crushed with a BFM-T6BI vibratory grinding and mixing machine (Jinan Beili Company, Jinan, Shandong) at 10 °C for 20 min. The ultra-micro powders were collected and tested for particle size. Among them, particles (D_50_ = 10.642 μm, particle size <75 μm) accounted for 92.9 % of all particles (19). According to the determination method of the “Pharmacopeia of the People's Republic of China” (2015 edition) (9), the content of polysaccharides was determined by the phenol-sulfuric acid method with glucose standard as the control substance. The content of crude polysaccharides in superfine powder of DO was 484.6 mg/g in the determination experiment (20). Subsequently, we weighed 10 g of the ultra-micro powder of DO and added them to 100 ml of sterile water. Then, the supernatant was heated and dissolved, cooled, and centrifuged at a low speed (3,000 r/min, 10 min) to prepare a concentration of 0.1 g/ml (21).

Lipid-lowering decoction is composed of six Chinese herbal medicines (22), including dried rhizoma of the *Alisma orinetale* (Sam.) Juzep. (10 g), dried seed of *Cassia obtusifolia* L. (30 g), dried rhizoma of the *Salvia miltiorrhiza* Bge. (10 g), dried rhizoma of the *Curcuma wenyujin* Y. H. Chen et C. Ling (10 g), dried algae of the *Sargassum pallidum* (Turn.) C. Ag. (30 g), and the dried leaf of the *Nelumbo nucifera* Gaertn. (10 g). The above herbs were purchased from the First Hospital of the Hunan University of Chinese Medicine. We first steeped the above Chinese herbal medicines in 10 times the volume of water for 30 min, brought them to a boil over high heat, and then simmered for 30 min over low heat, decocting twice. The two decoctions were mixed and concentrated and diluted to 0.17 g/ml of lipid-lowering decoction, stored at 4 °C.

### Animal care and experimental design

Twenty-four 4-week-old specific pathogen-free (SPF) Kunming mice (half male and half female), weighing 20 ± 2 g, were purchased from Hunan Slaccas Jingda Laboratory Animal Company (Hunan, China) with license number SCXK (Xiang) 2016-0002. All animals were raised in the SPF experimental animal room (constant temperature 23–25 °C, relative humidity 50–70%, and a 12-h light-dark cycle) of the laboratory animal center of the Hunan University of Chinese Medicine. The experimental protocols involved animals and their health care approved by the Institutional Animal Care and Use Committee of Hunan University of Chinese Medicine (SYXK (Xiang) 2019-0009) and were performed in strict accordance with the Guide for the Care and Use of Laboratory Animal of National Institute of Health (NIH) (Bethesda, MD, USA).

After 3 days of acclimation, 24 mice were randomly distributed into the normal saline-treated basal diet (bcn), normal saline-treated high-fat diet (bmn), 2.37 g kg^−1^ days^−1^ DO traditional decoction-treated high-fat diet (bdn), 1.19 g kg^−1^ days^−1^ lipid-lowering decoction-treated high-fat diet (bjn) groups, 0.35 ml per time, two times a day for 40 days. Six mice in each group. Female mice and male mice in the same group were raised separately housing (three male or three female mice per cage). We changed the bedding every 3 days and added water promptly. Among them, the basal diet contains 14% of energy from fat, 21% of energy from protein, and 64% of energy from carbohydrates. The high-fat diet contains 60% of energy from fat, 20% of energy from protein, and 20% of energy from carbohydrates (23).

### Observation of the general condition of mice

We observed and recorded the changes in hair, wet and dry feces, and mental status of the mice in each group. In addition, we checked the body weight of each group of mice on the 0th (original weight), the 7th day, the 14th day, the 21st day, the 28th day, and the 35th day of the experiment calculated the changes of body weight. ΔM_21_ = body weight of mice on the 7th day-body weight of mice on the 0th day; ΔM_31_ = body weight of mice on the 14th day-body weight of mice on the 0th day; ΔM_41_ = body weight of mice on the 21st day-body weight of mice on the 0th day; ΔM_51_ = body weight of mice on the 28th day-body weight of mice on the 0th day; ΔM_61_ = body weight of mice on the 35th day-body weight of mice on the 0th day.

### Collection and detection of blood samples

Mice in each group were sampled after fasting and dehydration for 12 h. After the blood was collected from the eyeballs, the blood was placed in the procoagulant tube for 20 min to separate the serum. Total cholesterol (TC), total triglyceride (TG), low density lipoprotein-cholesterol (LDL-C), and high density lipoprotein-cholesterol (HDL-C) were recorded by an automatic blood biochemical analyzer.

### Collection of intestinal contents samples

All mice were quickly executed by cervical dislocation, and the peritoneal cavity was immediately opened on an ultra-clean bench to remove the small intestine of the mice. Under aseptic conditions, the contents of the small intestine were taken with sterilized forceps. The sequencing samples were placed into 1.5 ml sterilized centrifuge tubes, numbered and weighed, and stored at −80°C for future research. In addition, to eliminate the difference between male and female mice, the intestinal contents of one male and one female mouse in each group were mixed into one sample.

### 16S rRNA gene high-throughput sequencing

PCR amplification was achieved using Q5 high-fidelity DNA polymerase (NEB Corporation); extracted DNA was used as a template to strictly control and minimize the number of amplification cycles, but maintain the same amplification conditions. Amplification was performed using the 16S rDNA V4 variable region, and the amplification products were detected by electrophoresis. For further fluorescence quantification, samples should be mixed in the appropriate proportions. Using the fluorescent quantitation method of Promega, the recycled products were quantitatively expanded based on the preliminary electrophoretic results; the samples were mixed in a corresponding proportion based on the fluorescent quantitation results. Afterward, the Illumina MiSeq platform was used to sequence. The procedure was: the sequencing library was prepared by Illumina in the following operational approach. Sequence end repair was performed, the bulged base of DNA sequence 5' end was removed by End Repair Mix2 in the kit, and a phosphate group was added to supplement the missing base of the 3' end. The target sequence was connected to the sequencing joint and fixed the DNA molecules on the flow cell. The self-connected fragment was removed, and DNA fragment was amplified by PCR. The library system after adding the joint was further selected and purified. Sequencing was completed by Wuhan Frasergen Genetic Information Company (Wuhan, China) (24).

### Bioinformatics and statistical analysis

The abundance of each Operational Taxonomic Unit (OTU) in each sample and the classification of these OTUs were represented in OTU tables. OTU-level alpha diversity indexes, such as Chaos 1, ACE, Shannon and Simpson indexes, were measured by MOTHUR (version v.1.30.1, https://www.mothur.org/) and visualized by GraphPad software. The Chaos 1 and ACE indexes focused on community richness (25), while the Shannon and Simpson indexes focus on community evenness (26, 27). The structural variation of microbial communities across samples was investigated by the Beta diversity, including UniFrac distance metrics Principle component analysis (PCA) and non-metric multidimensional scaling (NMDS). PCA analysis evaluated the similarity between samples based on Euclidean distances and did not take into account the possible interrelationships between the original variables. NMDS analysis was not influenced by the value of sample distances and only considers the size relationship between each other, which might give more stable sorting results for data with complex structures. LEfSe was an analytical method based on linear discriminant analysis of effect sizes, which essentially combines linear discriminant analysis with non-parametric Kruskal-Wallis and Wilcoxon rank sum tests to screen for key biomarkers (28). Functional analysis was performed by comparing the 16S rRNA gene sequence data from the Green gene database. The known gene functional mapping database of intestinal microbiota composition was “mapped” to predict the metabolic function of the bacterial microbiota.

All data in the experiments were expressed as mean ± standard error (SD). Statistical analysis was implemented using a one-way analysis of variance (ANOVA) followed by an LSD *post-hoc* test to determine the differences between groups. The results were considered significant when *p* < 0.05. Analyzes were performed using IBM SPSS Statistics 24.0 (IBM, Corporation, Armonk, New York, NY, USA).

## Results

### General condition of mice in each group

**Figure 2:**
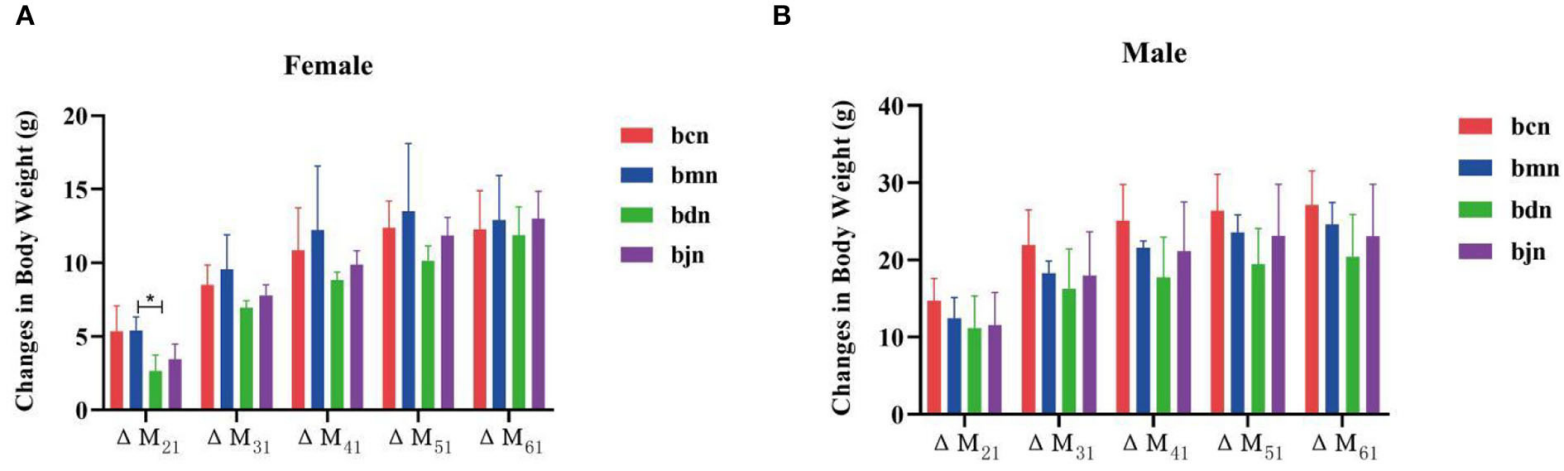
Effects of DO on the changes in body weight of mice fed high-fat diet. Data were mean ± SD, *n* = 6, ^*^p < 0.05. ΔM_21_ = body weight of mice on the 7th day-body weight of mice on the 0th day; ΔM_31_ = body weight of mice on the 14th day-body weight of mice on the 0th day; ΔM_41_ = body weight of mice on the 21th day-body weight of mice on the 0th day; ΔM_51_ = body weight of mice on the 28th day-body weight of mice on the 0th day; ΔM_61_ = body weight of mice on the 35th day-body weight of mice on the 0th day. bcn: normal saline-treated basal diet group; bmn: normal saline-treated high-fat diet group; bdn: DO traditional decoction-treated high-fat diet group; bjn: lipid-lowering decoction-treated high-fat diet group. **(A)** Weight changes of female mice. **(B)** Weight changes of male mice.

During the experiment, all mice had a normal appetite, activity, natural spirit, no difference in food consumption between groups, smooth and shiny hair, and unvaried tail length and thickness. Mice got along with each other harmoniously, with no abnormal phenomenon. A few mice had thin and wet feces every day, which is more common in female mice. However, the feces of some mice will become dry in the later stage. Figure 2A presented that the changes in body weight of female mice in the bmn group were all higher than those in the bcn group. The changes in body weight of female mice in the bjn group were all lower than those in the bmn group. After DO intervention, the changes in body weight of female mice in the bdn group were lower than those in the bmn group, with a significant difference in ΔM_21_ (*p* < 0.05). As shown in Figure 2B, the changes in body weight of male mice in the bmn group were higher than those in the bcn group. The bmn and bjn groups both showed lower changes in body weight compared to the bmn group, but the differences were not significant. Accordingly, it appeared that DO regulated body weight changes in mice on a high-fat diet to a certain extent and had a better effect on the control of body weight gain in female mice.

### Analysis of serum biochemical indexes of mice

Compared with the bcn group, the serum levels of TC, LDL-C and HDL-C in the bmn group tended to increase (*p* > 0.05; *p* > 0.05; *p* > 0.05), while the TG level decreased slightly (*p* > 0.05). After DO intervention, the serum levels of TC, LDL-C, and HDL-C increased in comparison with the bmn group (*p* > 0.05; *p* > 0.05; *p* > 0.05), but the TG level displayed marked reduction (*p* < 0.05). The changes of LDL-C, HDL-C, and TC levels in the bjn group were generally the same as that in the bdn group (*p* > 0.05; *p* > 0.05; *p* > 0.05), but the level of TG slightly decreased (*p* > 0.05) (Table 1). The above results implied that DO had regulating effects on lipid levels in mice fed with a high-fat diet.

**Table 1:** Effects of DO on serum biochemical indexes of mice with high-fat die (mmol/L).

**Groups**	**TC**	**TG**	**LDL-C**	**HDL-C**
bcn	3.08 ± 0.95	2.11 ± 0.62	2.20 ± 0.81	0.33 ± 0.08
bmn	3.31 ± 0.82	1.86 ± 0.23	2.28 ± 0.45	0.37 ± 0.07
bdn	3.35 ± 0.47	1.19 ± 0.09^*^	2.88 ± 0.60	0.46 ± 0.07
bjn	3.35 ± 0.66	1.33 ± 0.25	2.95 ± 0.49	0.48 ± 0.10

Data were mean ± SD,

* p < 0.05 vs. bmn.

TC, Total cholesterol; TG, total triacylglycerol; LDL-C, low density lipoprotein-cholesterol; HDL-C, high density lipoprotein-cholesterol. bcn, normal saline-treated basal diet group; bmn, normal saline-treated high-fat diet group; bdn, DO traditional decoction-treated high-fat diet group; bjn, lipid-lowering decoction-treated high-fat diet group.

### Sequencing data quality assessment of intestinal contents microbiota

**Figure 3:**
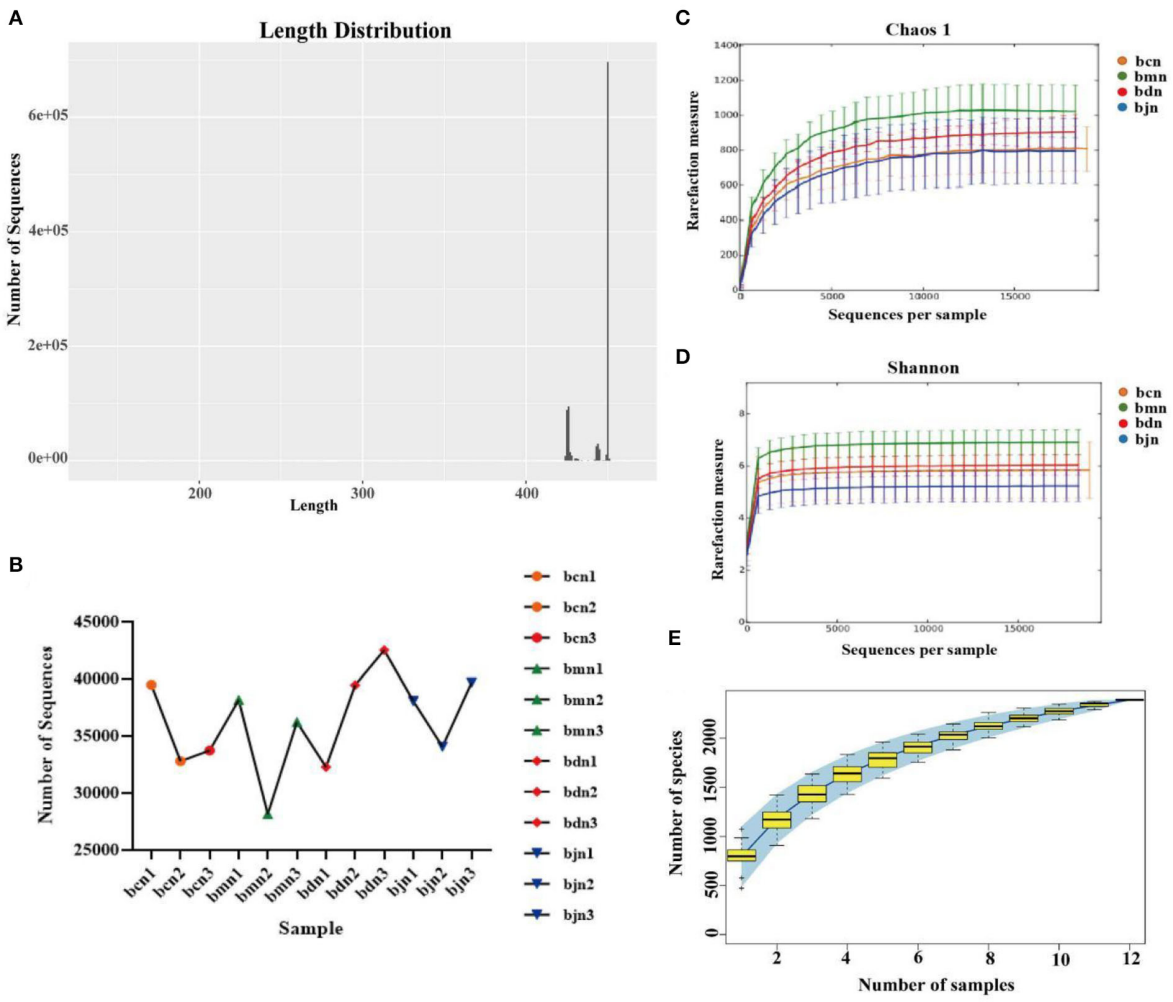
Sequencing data quality assessment of intestinal contents microbiota. **(A)** Sequence length distribution diagram. **(B)** The effective sequence quantity of each sample. **(C)** Dilution curve of Chaos 1. **(D)** Dilution curve of Shannon. **(E)** Species accumulation curve. bcn: normal saline-treated basal diet group; bmn: normal saline-treated high-fat diet group; bdn: DO traditional decoction-treated high-fat diet group; bjn: lipid-lowering decoction-treated high-fat diet group.

According to the statistics on the length of sequences obtained by this sequencing, the length distribution of sequences obtained by sequencing of each sample was concentrated in the vicinity of 400~500 bp (Figure 3A). After quality control, a total of 434,590 pieces of original sequencing data were obtained (Figure 3B). The dilution curve showed that when the sequence amount of each sample reached 5,000, the curve entered the plateau phase, and the microbial biomass detected in each sample approached saturation (Figures 3C,D), indicating that the current sequencing depth was sufficient to reflect the microbial diversity contained in this batch of samples. As shown in Figure 3E, the samples continued to increase, the rate of increase in OTU number slowed down and the curve tended to flatten, demonstrating that with the addition of new samples, the total number of OTUs almost did not increase, which proved that the sample of this study was sufficient to meet the needs of the study (Figure 3E). So, we hypothesized that a reasonable sequencing depth was used in this study and that the amount of data sequenced from the samples was sufficient to adequately represent the real appearance of the microbial community in each sample and could be used for the microbial diversity analysis of this batch of samples.

### OTU numbers and diversity of intestinal contents microbiota

**Figure 4:**
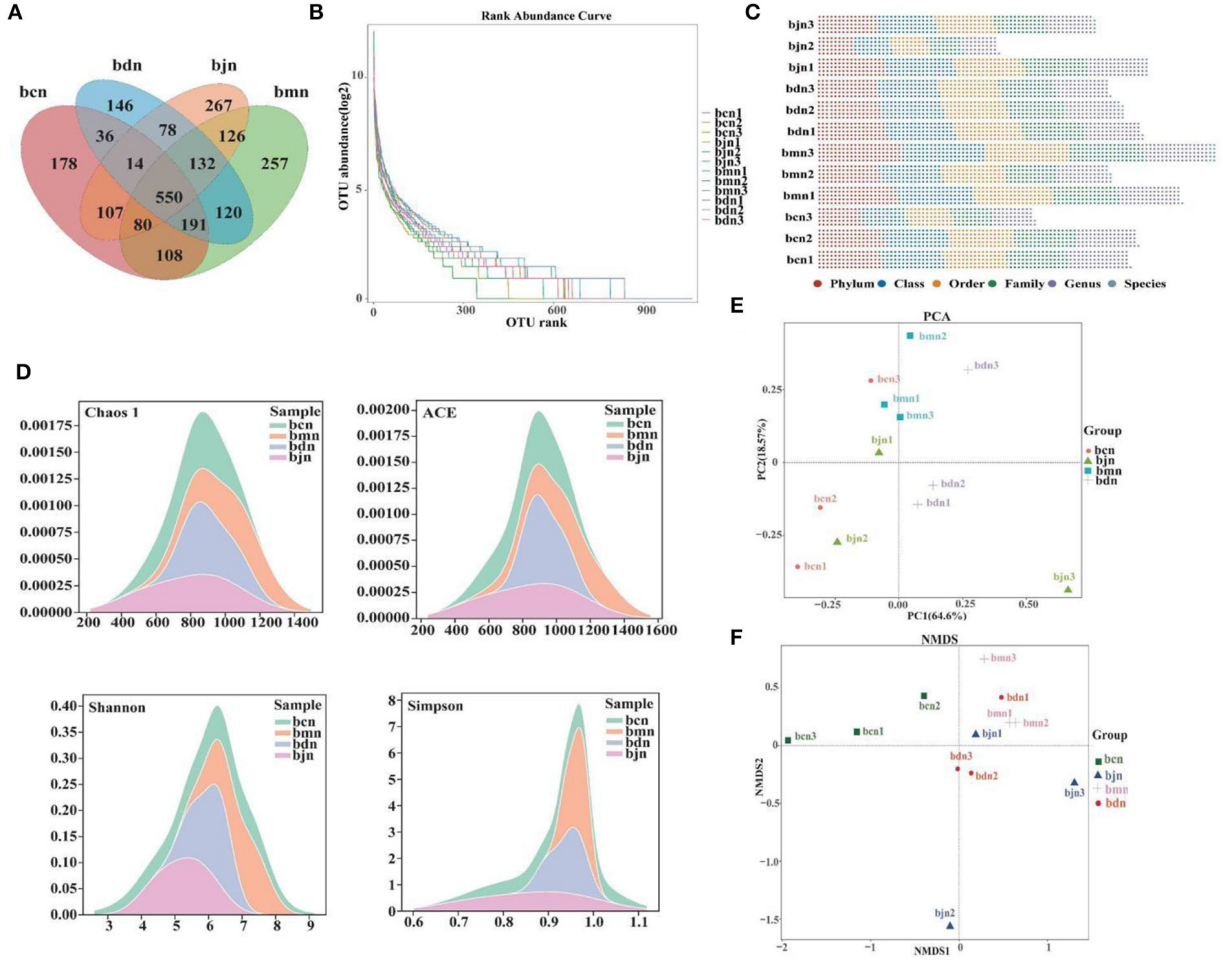
OTU number and diversity of intestinal contents microbiota. **(A)** Venn diagram. **(B)** Rank abundance distribution curve. The longer the folded line, the greater the number of OTUs in the sample. The gentler the curve, the better the uniformity of the community; the steeper the curve, the lower the homogeneity of the community. **(D)** OTU number of each sample at different classification levels. **(C)** OTU in each sample at each classification level. **(D)** Chaos 1 index; ACE index; Shannon index; Simpson index. **(E)** PCA analysis. **(F)** NMDS analysis. Data were mean ± SD, *n* = 3, *p* > 0.05. bcn: normal saline-treated basal diet group; bmn: normal saline-treated high-fat diet group; bdn: DO traditional decoction-treated high-fat diet group; bjn: lipid-lowering decoction-treated high-fat diet group.

The total OTUs in the bcn, bmn, bjn, and bdn groups were 1,264, 1,564, 1,354, and 1,267, respectively. The number of OTUs in the intersection of the four groups was 550 (Figure 4A). The rank abundance distribution curve was drawn based on abundance log2 values (Figure 4B). We found that the number of OTU was most enriched in the bmn group, which was in agreement with the Venn diagram reported above. OTU numbers of each sample in different classification levels were different (Figure 4C). The Chaos 1, ACE, Shannon and Simpson indexes in the bmn group had a mild increase in comparison with the bcn group (Figure 4D). In the bdn group, the above four indexes had a decreased trend compared with that in the bmn group, reflecting that intervention with DO regulated the diversity of intestinal microbiota to a certain extent. The samples from the bcn group were located in the first and third quadrants and were relatively concentrated. Samples of the bmn group were located in the first and second quadrants. The bdn group was separated from the bmn group, with the bjn and bcn groups distributed in between (Figure 4E). NMDS results indicated that there was a clear separation between the bmn and bcn groups (Figure 4F). The above results demonstrated that DO intervention decreased the richness and diversity of intestinal contents microbiota, and altered the structure as a whole.

### Dominant bacteria and differential bacteria of intestinal contents microbiota

**Figure 5:**
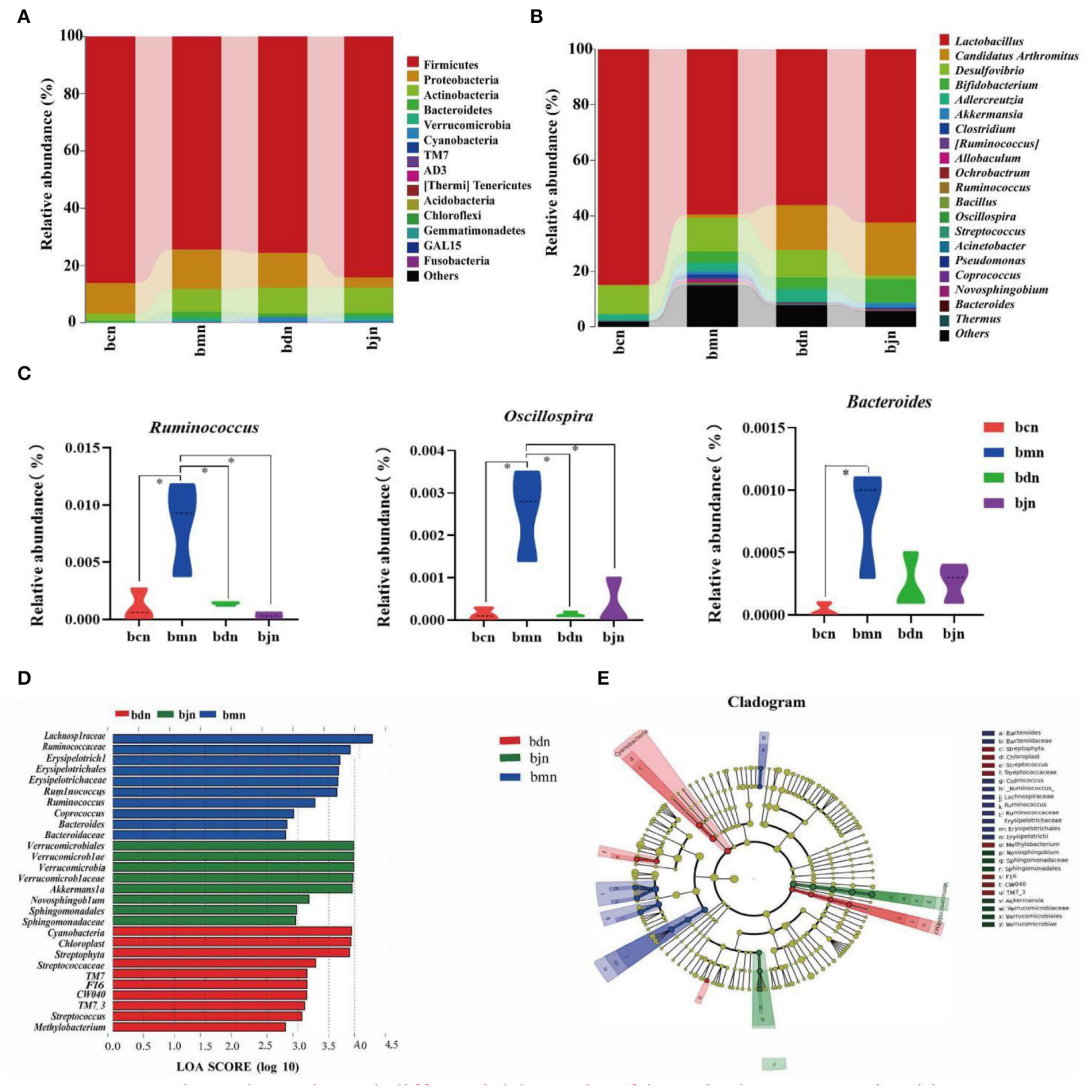
Dominant bacteria and differential bacteria of intestinal contents microbiota. Data were mean ± SD, *n* = 3, ^*^p < 0.05. **(A)** Intestinal contents microbiota composition in the phylum level. **(B)** Intestinal contents microbiota composition in the genus level. **(C)** Genus levels of dominant bacteria. **(D)** LDA scores plot. The horizontal coordinates were the logarithmic scores of LDA for each classification unit. The vertical coordinates were the classification units with significant differences between groups. The longer the length, the more significant the difference was. **(E)** Cladogram diagram. The circles radiating from the inner to the outer layers of the diagram represented the taxonomic hierarchy of species from phylum to species. Nodes indicated a taxonomic unit at the taxonomic level. Letters identified the names of taxonomic units with significant differences between groups. bcn: normal saline-treated basal diet group; bmn: normal saline-treated high-fat diet group; bdn: DO traditional decoction-treated high-fat diet group; bjn: lipid-lowering decoction-treated high-fat diet group.

We screened the top 20 phyla and genera in terms of relative abundance and presented them in bar charts (Figures 5A,B). Then, statistical analysis of the above species revealed (Figure 5C) that, compared to the bcn group, the bmn group had a notably higher abundance of *Ruminococcus, Oscillospira*, and *Bacteroides* (*p* < 0.05; *p* < 0.05; *p* < 0.05). The bdn group had a substantial reduction of *Ruminococcus* and *Oscillospira* compared with the bmn group (*p* < 0.05; *p* < 0.05). Therefore, DO intervention inhibited the growth of dominant bacteria including *Ruminococcus* and *Oscillospira*. LEfSe analysis results presented that 10, 10, and 8 dominant taxa in the bmn, bdn, and bjn groups, respectively (Figure 5D). Among them, the bmn group was markedly enhanced in genera *Bacteroides, Ruminococcus*, and *Coprococcus*. The bdn group was rich in genera *Streptococcaceae, Methylobacterium*, and *Streptococcus*. The bjn group was characterized by a greater increase in the abundance of genera *Akkermansia, Sphingomonadaceae, Novosphingobium*, and *Verrucomicrobiaceae*. In the different classification systems (Figure 5E), there were significant differences in the differential bacteria among the three groups.

### Functional analysis of intestinal contents microbiota

**Figure 6:**
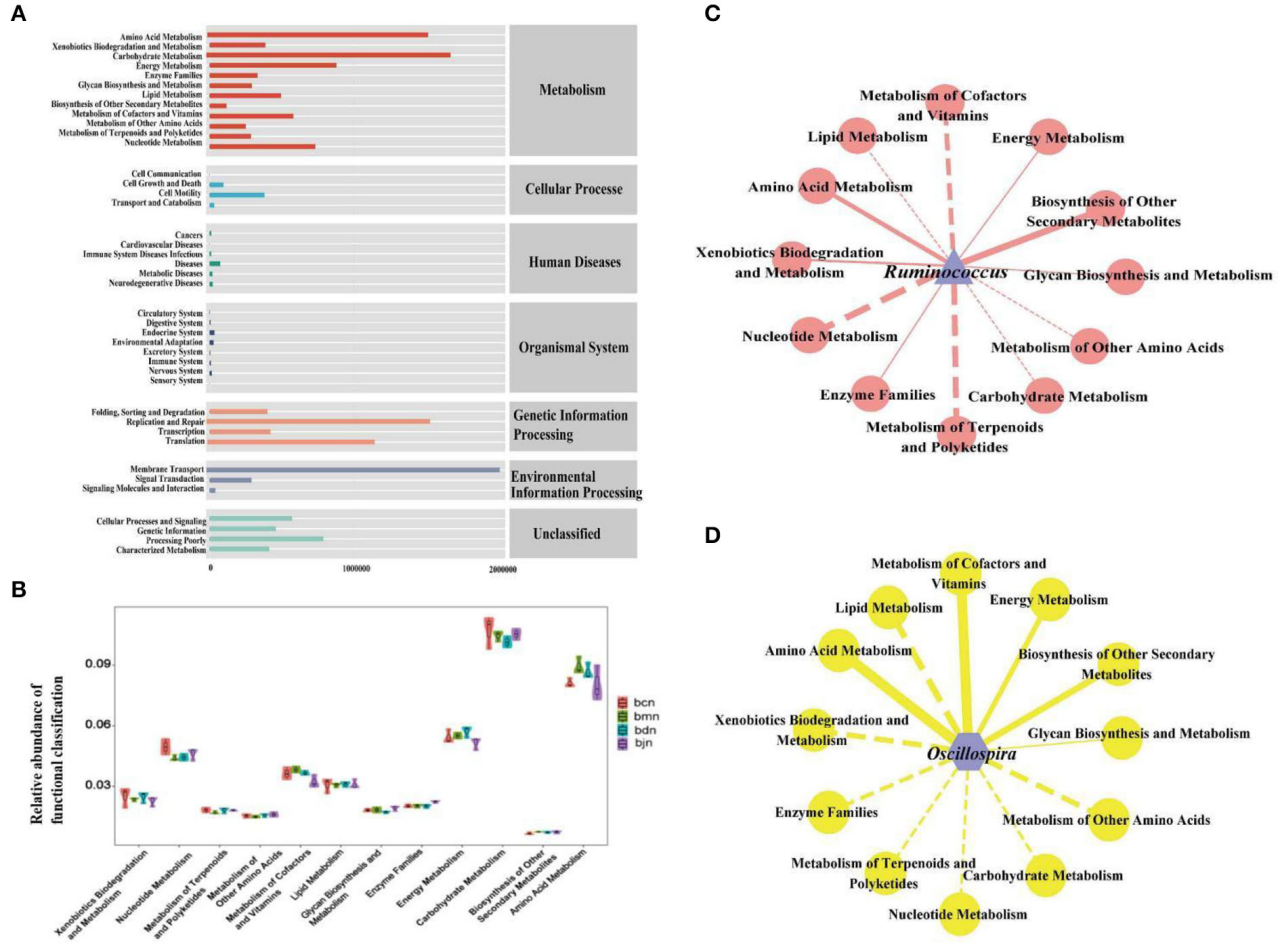
Functional analysis of intestinal contents microbiota. **(A)** Predicted abundance of KEGG function. **(B)** Histogram of metabolic function. **(C)** Interaction network of “*Ruminococcus*-metabolic function”. **(D)** Interaction network of “Oscillospira-metabolic function.” Solid line represented positive correlation, dotted line represented negative correlation. The thickness of the line indicated the strength of the correlation. bcn: normal saline-treated basal diet group; bmn: normal saline-treated high-fat diet group; bdn: DO traditional decoction-treated high-fat diet group; bjn: lipid-lowering decoction-treated high-fat diet group.

The intestinal contents microbiota function was generally divided into seven categories, and the second level included 41 sub-functional categories, with the metabolic function accounting for a greater abundance (Figure 6A). As shown in Figure 6B, it had a great influence on carbohydrate metabolism, amino acid metabolism and energy metabolism. Compared with the bcn group, the metabolic pathways were altered in the high-fat diet, including a decrease in carbohydrate metabolism and amino acid metabolism and an increase in energy metabolism. After DO intervention, the carbohydrate metabolism and amino acid metabolism decreased, while energy metabolism increased. Compared to other metabolic functions (Figures 6C,D), *Ruminococcus* and *Oscillospira* had a stronger positive regulation effect on amino acid metabolism and biosynthesis of other secondary metabolites, and a stronger negative regulation effect on the metabolism of terpenoids and polyketides.

### Correlation analysis

**Figure 7:**
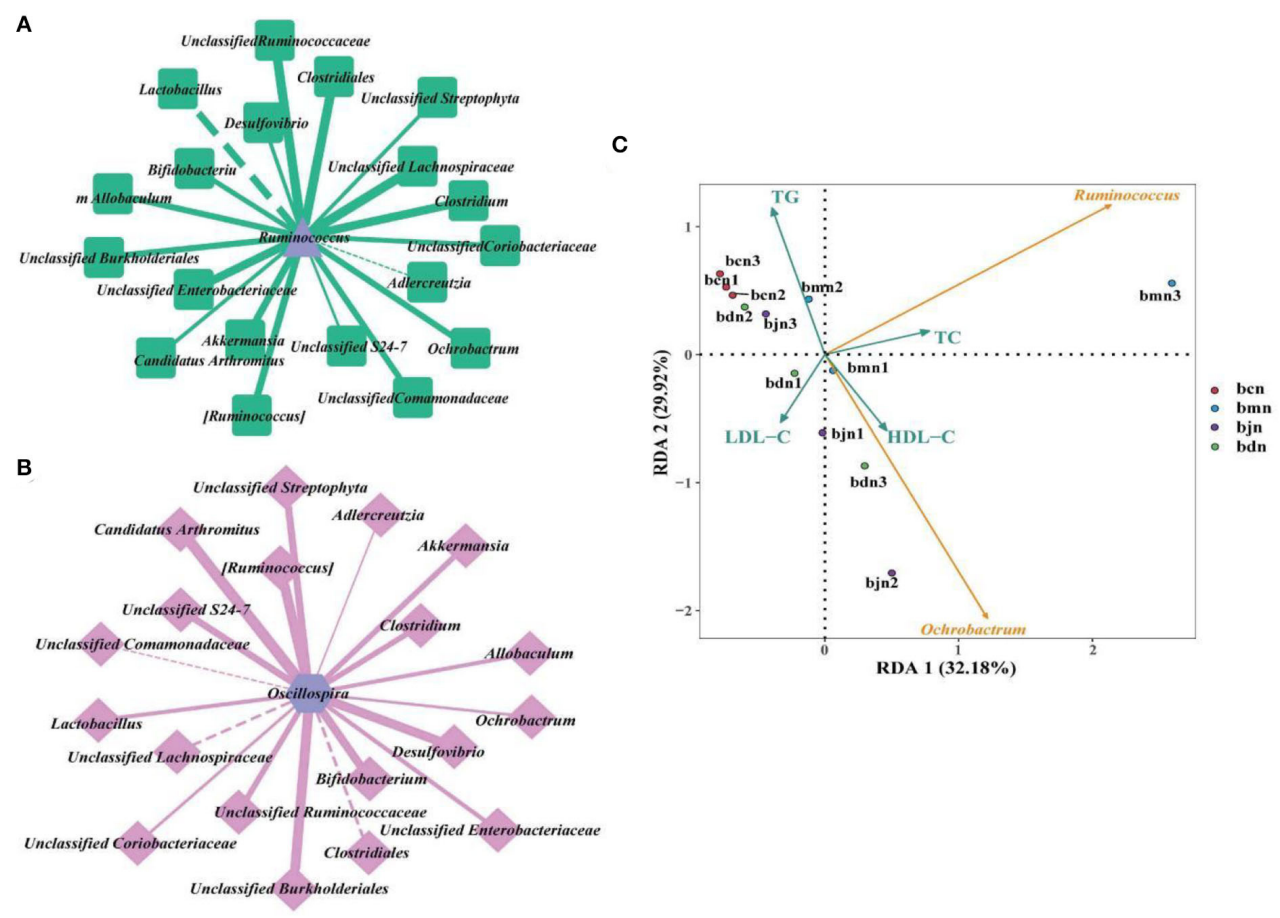
Correlation analysis. **(A)** “*Ruminococcus*-dominant bacteria” interaction network at the genus level. **(B)** “*Ochrobactrum*-dominant bacteria” interaction network at the genus level. Solid line represented positive correlation, dotted line represented negative correlation. The thickness of the line indicated the strength of the correlation. **(C)** RDA analysis. Different colored arrows represented the dominant bacteria and environmental factors. Dots of different colors represented samples from different groups. The Angle between the arrow lines represented correlation, an acute angle represented positive correlation, and an obtuse angle represented negative correlation. bcn: normal saline-treated basal diet group; bmn: normal saline-treated high-fat diet group; bdn: DO traditional decoction-treated high-fat diet group; bjn: lipid-lowering decoction-treated high-fat diet group.

Combined with correlation coefficient analysis, we constructed interaction networks of “*Ruminococcus*- dominant bacteria” and “*Oscillospira*- dominant bacteria,” respectively (Figures 7A,B). The results presented that *Ruminococcus* and *Oscillospira* showed positive regulatory effects with most genera. Therefore, we hypothesized that interactions between dominant genera might be responsible for the changes in intestinal contents microbiota in mice on a high-fat diet with DO intervention. Subsequently, We performed redundant analysis (RDA) to explore the relationship between blood biochemical indicators and *Ruminococcus, Oscillospira* (Figure 7C). The results showed that *Ruminococcus* was positively correlated with TC, TG, and HDL-C, and negatively correlated with LDL-C. *Oscillospira* was positively correlated with TC, LDL-C, and HDL-C, and negatively correlated with TG. It was suggested that the interaction between the above factors might be involved in the regulation of intestinal contents microbiota in mice on a high-fat diet by DO.

## Discussion

The human intestinal microbiome, a diverse and complex ecosystem, is home to thousands of microorganisms that have co-evolved with their hosts and play a weighty role in health and diseases. The composition of the intestinal microbiome varies from person to person and at different stages of host development and is dependent on host genotype and environmental factors (29). The intestine of a young mammal is sterile in the mother, and its intestinal microbiota comes mainly from maternal colonization and acquired intake (30). Among them, diet is considered to be the main factor influencing the structure of the intestinal microbiota and plays a key role in the colonization, maturation, and stabilization of the intestinal microbiota (31). Studies have indicated that the corresponding microbial environment of the intestinal microbiota of mice could be induced within 1 day after dietary modification (32). Wang et al. found that a high-fat diet caused changes in the composition of the intestinal microbiota of mice, with more pronounced changes in the Firmicutes, Proteobacteria, and Bacteroidetes (33). In our experiment, the structure and composition of intestinal contents microbiota were changed in mice following the high-fat diet. Compared with the bcn group, the bmn group showed an increase in the number of OTUs as well as a change in diversity and richness. The bacterial composition of the intestinal contents microbiota of the model mice showed changes at all taxonomic levels, with the *Ruminococcus, Oscillospira*, and *Bacteroides* changing more significantly. The above results insinuated that a high-fat diet changed the intestinal microbial community to a certain extent, which was in line with the previous studies that demonstrate differences in the composition and structure of intestinal microbiota in the occurrence of hyperlipidemia (34).

Modern medical research indicated (35–37) that DO were rich in polysaccharides, alkaloids, amino acids, and other active ingredients, which had excellent hypoglycemic, antioxidant, and immune boosting properties. Modern studies verified its diverse intestinal health functions. Domestic and international research on DO had not only focused on its pharmacological effects and health functions but on the safety of DO had received increasing attention. The current research reported that DO and its series products were not found to be toxic in terms of acute toxicity (38), genotoxicity (39), reproductive toxicity (40), or teratogenic toxicity (41) and could be safely taken. In 2020, the National Health Commission of the People's Republic of China issued the pilot project that carried out the production and operation of nine substances, including DO, which were traditionally both food and herbal medicines. It also brought an opportunity for the development of the DO. In this experiment, we analyzed the effect of DO on the changes in body weight of mice on a high-fat diet and confirmed that DO inhibited the body weight increase of mice on a high-fat diet to a certain extent, and there were differences in the regulation of body weight of mice of different sexes. Previous studies had reported (42) that the rich polysaccharide contents of DO had a positive effect on weight gain control, which was consistent with the results of our experiments. In addition, gender differences in lipid metabolism, and this difference might be related to differences in the amount of lipid substances in muscle tissue. The oxidative metabolism of lipid substances in muscle tissue required the involvement of relevant hormones (43, 44). Hence, we hypothesized that differences in energy metabolism in males and females might be one of the reasons for the differences in body weight regulation by DO in mice on high-fat diets of different sexes.

We investigated the effects of DO on the diversity, community structure, and function of intestinal contents microbiota in mice fed with a high-fat diet. Alpha diversity indexes of the bdn group were lower than that of the bmn group. Additionally, the diversity indexes of the bdn and bjn groups were closer to that of the bcn group, suggesting that DO restored the diversity to normal levels. According to the analysis of Beta diversity, it was implied that DO improve the chances of the community structure of the intestinal contents microbiota in mice induced by a high-fat diet, and gradually restored to the normal level. However, there were still discrete individual samples in the bdn group, which might be caused by the differences between individual mice as well. These results seem to be consistent with a previous study that the biodiversity of mice intestinal microbiota was elevated and the community similarity coefficients of intestinal microorganisms showed significant differences after the intervention by the DO aqueous extract (17). Taken together, we hypothesized that DO had the effect of regulating the diversity and changes in microbial communities in mice induced by a high-fat diet. By comparing the relative abundance in the bdn group and bmn group, we further understood how DO changes the intestinal microbial environment. At the genus level, it was worth noting that the relative abundances of *Ruminococcus* and *Oscillospira* in the bdn group were sharply decreased. Studies have illustrated the role of these bacteria in intestinal health. *Ruminococcus* was often closely related to the adverse effects associated with a high-fat diet by positively regulating the occurrence of obesity (45, 46). Researchers confirmed the protective effect of *Ruminococcus* on long-term weight gain in a longitudinal study of the intestinal microbiota in healthy women (47). All of the above suggested that diet-induced changes in the composition of the intestinal microbiota, to a certain extent, affected the metabolic function of the host, leading to weight gain. Furthermore, *Ruminococcus* played a vital role in the lipids metabolism process. We improved the adverse effects of a high-fat diet by reducing the contents of *Ruminococcus*, which was partly achieved by DO. Large amounts of *Oscillospira* were associated with systemic inflammation and changes in intestinal permeability. And the study found that *Oscillospira* induced high-fat diet-related hepatic steatosis (48). Amandine et al. illustrated that *Oscillospira* was positively correlated with the obesity index (49). Similar results were likewise manifested in our results, suggesting that DO might produce anti-hyperlipemia effects in mice fed with a high-fat diet by reversing the abundance of *Oscillospira*. In the LEfSe analysis, there were critical differences in the characteristic microbiota among the bmn group, bdn group, and bjn group, further proving the differences in intestinal contents microbiota structure among the above three groups. Subsequently, DO might intervene with the therapeutic process *via* multiple pathways such as carbohydrate metabolism and amino acid metabolism in mice based on functional analysis of the metabolic pathways. Moreover, intestinal contents microbiota has a certain regulatory effect on TC, TG, LDL-D, and HDL-D (50). It was found that Ruminococcus could achieve positive regulatory effects on TC and HDL-D, while LDL-C showed mostly negative regulatory effects. Also, Oscillospira was negatively correlated with TG (51–53). These results were similar to the RDA results presented in our experiment. Therefore, we hypothesized that the *Ruminococcus* and *Oscillospira* might be one of the factors affecting the changes in serum levels of TC, TG, LDL-C, and HDL-C in mice during the high-fat diet and DO intervention.

The influence of gender factors on intestinal microbiota was also a point worth paying attention to in this experiment. Sinha et al. conducted sex-specific analyses of fecal microbiota composition in 1,135 men and women and indicated that there were significant differences in the overall gut microbiome composition of men and women (54). Peng et al. found that a high-fat diet had different effects on intestinal microbiota in female and male mice. Compared with the control diet group, the relative abundance of Proteobacteria phylum in the female high-fat diet group significantly decreased, while the Actinobacteria markedly increased. However, the Proteobacteria in the male high-fat diet group significantly increased and the Actinobacteria had no obvious change. Moreover, the relative abundance of *L.murinus* was markedly higher in the high-fat diet group than that in the control diet group of female mice. This species of bacteria did not show a significant change in the male high-fat diet group (55). In our experiment, we mixed the intestinal contents of one male and one female mouse in each group into one sample during the collection of intestinal contents samples in order to minimize the influence of gender differences in the process of intestinal microbiota examination. However, this same procedure had also been reported in the experiments of Chen et al. (56) and Li et al. (57).

The lipid-lowering decoction was the clinical experience prescription of national famous Chinese medicine professor Cheng et al. (58). In the clinical treatment of non-alcoholic steatohepatitis, it had been found that lipid-lowering decoction played the effect of regulating lipids, protecting the liver, and reducing weight, which had a clinical efficiency of 88%. It was effective in reducing blood lipid levels and liver function indicators without significant toxic side effects (59). Animal experiments made the point that lipid-lowering decoction significantly reduced the levels of lipid, liver fat, and free fatty acids in the blood of rats with non-alcoholic fatty liver disease caused by the high-fat diet. It reduced free fatty acid contents, inhibited triglyceride and cholesterol deposition in the liver, and effectively improved lipid metabolism, thus achieving a lipid-lowering effect (60). In our experiment, lipid-lowering decoction, as a positive control, inhibited the body weight change rate and expressions of TC, TG, and LDL-C in the serum of mice on a high-fat diet, decreased the Chaos 1, ACE, Shannon, and Simpson indexes of the intestinal contents microbiota, regulated the differences in the community structure and composition, reduced the amino acid metabolism and energy metabolism and improved the carbohydrate metabolism. It was evidenced that lipid-lowering decoction played a role in regulating body weight, lipid metabolism, and the structure and function of intestinal contents microbiota in mice on a high-fat diet.

In summary, the diversity and richness of intestinal microbiota, as well as microbial interactions appear to be key factors in the regulation of intestinal health. This study revealed that DO had a moderating effect on diversity, community structure, and functions of intestinal contents microbiota in mice fed with high-fat diet. We speculated that the mechanism of DO against high-fat diet diseases might be attributed to the inhibition of *Ruminococcus* and *Oscillospira*, leading to a promotion in the state of host health. In the next steps, we need to further understand the interactions of intestinal microorganisms (including synergistic metabolism, antagonistic competition, etc.) and the correlation analysis between intestinal microorganisms and the active ingredients in DO, which will also provide a basis for targeting DO to regulate intestinal micro-ecological in the future.

## Data availability statement

The datasets presented in this study can be found in online repositories. The names of the repository/repositories and accession number(s) can be found at: NCBI; PRJNA854391.

## Ethics statement

The animal study was reviewed and approved by The Institutional Animal Care and Use Committed of Hunan University of Chinese Medicine [SYXK (Xiang) 2019-0009].

## Author contributions

XL prepared the drafting of manuscript, analysis, and interpretation of data. ND and TZ analyzed in the analyzing of part of the data. BQ and MP performed the experiment. NX and ZT revised the initial manuscript critically. All authors contributed to manuscript revision, read, and approved the submitted version.

## Funding

This study was supported by the Hunan province double first-class discipline of Traditional Chinese medicine.

## Conflict of interest

The authors declare that the research was conducted in the absence of any commercial or financial relationships that could be construed as a potential conflict of interest.

## Publisher's note

All claims expressed in this article are solely those of the authors and do not necessarily represent those of their affiliated organizations, or those of the publisher, the editors and the reviewers. Any product that may be evaluated in this article, or claim that may be made by its manufacturer, is not guaranteed or endorsed by the publisher.

## Author disclaimer

The views expressed herein are solely those of the authors and do not represent the official policies or positions of any supporting agencies.
